# Rethinking labour migration: Covid-19, essential work, and systemic resilience

**DOI:** 10.1186/s40878-021-00252-2

**Published:** 2021-09-30

**Authors:** Bridget Anderson, Friedrich Poeschel, Martin Ruhs

**Affiliations:** 1 Migration Mobilities Bristol, University of Bristol, 11 Priory Road, Bristol, BS8 1TU UK; 2 Migration Policy Centre, Robert Schuman Centre for Advanced Studies, European University Institute, via Boccaccio 151, 50133 Florence, Italy; 3 Department for Continuing Education, University of Oxford, Rewley House, 1 Wellington Square, Oxford, OX1 2JA UK

**Keywords:** Covid-19, Essential services, Systemic resilience, Migrant workers, Labour migration policies

## Abstract

Many of the ‘essential workers’ during the Covid-19 pandemic are migrants, playing an important role for the continued functioning of basic services – notably health services, social care, and food supply chains. We argue that this role should be taken into account when assessing the impacts of migrant workers and in the design of labour migration and related public policies. Existing studies highlight how the employment of migrant workers in essential services is shaped by interests of employers, sectoral policies, and national institutions. Considerations of how migrants may affect the systemic resilience of essential services – in a pandemic or similar crises – are pervasively absent, not only in policy-making but also in research. Drawing on several disciplines, we outline the concept of systemic resilience and develop implications for the analysis and regulation of labour migration. We call for shifting the focus from the role of migrants in specific occupations and sectors in particular countries to transnational systems of production and service provision. To study how migrant workers affect systemic resilience, we propose an agenda for comparative research along three lines: comparing migrants to citizens within the same system, comparing migrants’ roles across systems, and comparing strategies for resilience adopted in different systems.

## Introduction

In the spring and summer of 2020, the Covid-19 pandemic and associated bans on movement within and across countries led to severe labour market shocks. These included both sharp increases in labour demand (e.g. in the health sector) and reductions in labour supply (e.g. in agriculture and the care sector). In this global health emergency, the protection and maintenance of essential economic activities and public services has been a central policy challenge. Essential activities and services include sectors such as agriculture and food production, health services and social care, as well as key digital and non-digital infrastructures such as transport and logistics. Thus, Covid-19 raised and continues to raise urgent questions for research and policy about the factors that affect the (lack of) resilience of the provision of essential goods and services during both what is, at time of writing, the ongoing pandemic, and also anticipated future health shocks.

Following the outbreak of Covid-19, governments around the world instituted a range of restrictions on movement and access to workspaces. These were imposed at different times depending on the pandemic phase but between March and June of 2020, the shutdown affected most parts of the world. However, some jobs were declared ‘essential’, exempting them from the most severe restrictions; indeed in some cases governments even required certain workers to go to work. In many countries essential jobs were identified through lists published by the central government (e.g. Italy, Spain, Mozambique, Brazil and the United Kingdom) or at state level (e.g. Germany and the United States). These lists varied considerably in form and level of detail, but there were some goods and services that were common to lists across countries and states, including food provision, health and some care services.

In many high-income countries, ‘migrants’ account for substantial shares of employment in sectors designated essential and therefore they often represented a substantial proportion of those designated ‘essential workers’ or ‘key workers’ (e.g. Fasani & Mazza, 2020; Fernández-Reino et al., 2020; Gelatt, 2020; OECD, 2020a). In this context, by ‘migrant’ we mean non-citizens who are subject to immigration controls, or who may be legitimately refused entry in exceptional circumstances (e.g. EU citizens, who are not subject to immigration controls but whose free movement between EU member states was curtailed during pandemic restrictions). Essential workers included migrants typically considered ‘low-skilled’ such as crop pickers, food processors, care assistants, and cleaners in hospitals. States in the Global North sought to protect, and in some case even expand the supply of such workers during the health emergency. For example: the Italian government granted temporary legal status to migrants employed irregularly in agriculture and the care sector in spring 2020; the United Kingdom announced the automatic extension of visas of migrant doctors, nurses, and paramedics; Austria and Germany exempted migrants working on farms and in care homes from international travel bans; in the United States, while normal consular operations were suspended, foreign farm workers were still permitted to apply for and receive work visas (OECD, 2020a). Such measures were widely accepted as pragmatic responses to Covid-19, but they raise important questions about the role of migrants in national labour markets. Whether, why, and to what extent migrant workers are ‘needed’ to reduce labour and skills shortages in essential services are questions that have been the subject of research in recent years. What Covid-19 invites us to consider is migrants’ role in ensuring longer-term *resilience* of essential services.

Resilience may be broadly defined as the ability to withstand, recover from, and adapt to unexpected external shocks (OECD, 2020b). It has featured in some aspects of public administration and policy, including military and public health matters, but the role of migration and migrants in the resilience of essential economic activities and services has remained unexplored. Moreover, research on demand for migrant labour has tended to focus on employers’ incentives rather than the essential nature of these sectors or the effects that prioritising *systemic resilience* might have on the demand for migrant workers. Indeed, with the exception of the resilience of destination country institutions in the case of irregular migration flows (Geddes, 2015; Paul & Roos, 2019), research on migration and migration policy has not drawn on the concept of resilience, in contrast with many other research areas (Bourbeau, 2015).

This paper addresses this gap by outlining a theoretical basis for a new research agenda that can help inform policy debates about the role of migrant workers in the provision and resilience of essential services during the current pandemic and similar future shocks. It analyses why and how a concern for the resilience of essential services can help us rethink the ways of assessing impacts of migrant workers. This in turn can feed into the design of labour migration and related public policies. The paper combines key insights from research on the role of migrant workers in addressing labour and skills shortages (Section 2) with the (essentially disconnected) studies of systemic resilience (Section 3) to suggest how considerations of systemic resilience can be integrated into analyses and policy debates on labour migration (Section 4). Emphasising the need for a transnational perspective, the discussion considers migrants not only in essential services of a single country but also along global supply chains. In addition, due to different institutions and policies across countries, considerable variation can be expected in terms of politics, governance, and the strategies used to pursue resilience. Our discussion of key elements of a future research agenda on how migrants can shape systemic resilience (Section 5) thus highlights the importance of comparative institutional analysis that takes a transnational approach.

## Past learning: a need for migrant workers?

In the past, the terminology of ‘essential work’ and ‘key industries’ has been used to refer to domestic production that exempts workers from conscription, e.g. the UK Essential Work (General Provisions) Order of March 5, 1941. The topic of migrants as essential workers has emerged only with the outbreak of Covid-19. However, the literature has long debated to what extent migrants are ‘needed’ for addressing shortages in the supply of labour and skills, usually in specific sectors and occupations. Employers and other stakeholders often argue that migrant workers are needed because certain jobs cannot be filled otherwise. Sceptics suspect that employers seek to avoid raising wages or improving employment conditions and prefer recruiting exploitable migrant workers. Several insights from this debate appear useful for a discussion on migrants as essential workers, in particular the research findings on employers’ demand for migrant labour, the characteristics and determinants of labour and skills shortages, and the alternative policies for responding to them (e.g. Ruhs & Anderson, 2010; Waldinger & Lichter, 2003).

Firstly, with regards to labour or skills ‘shortages’, there is no universally accepted definition nor one ‘optimal’ policy response. In the context of employers’ calls for migrant workers, ‘labour shortage’ typically refers to the demand for labour exceeding supply at prevailing wages and employment conditions. Some employers may be reluctant or unable to respond by offering higher wages. This exemplifies the central role of wages, employment conditions and structural constraints for debates about labour shortages. Such considerations likely also apply in the context of essential services: for example, employment conditions can simultaneously create flexibility for employers (which might contribute to resilience) and precarity for workers (which might undermine resilience).

The definition of ‘skills’ is similarly contentious, which complicates analyses and debates about ‘skills shortages’. What is recognised and legitimated as ‘skill’ is socially constituted, unavoidably politicised, and often heavily gendered (Bryant & Jaworski, 2011; Fenwick, 2006; Guo, 2015; Sawchuk, 2008; Steinberg, 1990). Immigration schemes mostly focus on credentialised skills, based on formal educational qualifications and measured through earnings. However, the skills that matter for employers likely vary considerably by country, company, and job profile (Griuglis & Vincent, 2009). Foreign qualifications are often not directly transferable, due to country-specific licenses and the need to be complemented by language skills (e.g. Chiswick & Miller, 2003). In addition, the notion of skills used by immigration schemes does not always adequately capture ‘soft skills’, which include important employee qualities such as attitude, presentation, social interaction etc. (Sawchuk, 2008). In some occupations, soft skills like ‘work ethic’ demanded by employers partly or largely reflect employer preference for workers who accept particular mechanisms of control and/or wages and employment conditions that are not sufficiently attractive for local workers.

In some states the Covid-19 pandemic prompted public acknowledgement that ‘low-skilled’ workers were providing services critical to social and economic functioning. The pursuit of resilience as a desirable goal might therefore change how the socio-economic contributions of low-skilled or unskilled workers are valued and analysed. It may also require rethinking how skill is understood in practice, particularly the tendency to assume an association between skill and specialisation. This association shapes understandings of the nature of skill with workers who have a large number of generalised competences and are often labelled ‘low-skilled’. With regards to the recognition of foreign qualifications, the pandemic has already led to policy changes: Colombia and Peru, for example, simplified the recognition of foreign medical qualifications to allow for recruiting Venezuelan refugees (Freier & Luzes, 2020).

Previous research has also found that one reason employers seek to employ migrant workers is the additional means of control over migrants extended by immigration requirements, as workers are often not free to leave sponsoring employers (e.g. Anderson, 2010). This highlights that immigration controls are not neutral ‘taps’ allowing in the necessary number of workers, but actively shape employment relationships and rights, creating a labour force with often quite specific constraints that differentiate them from citizens. During the pandemic, for example, some US employers saw migrant workers as less likely to become infected, because they were assigned to on-site accommodation and discouraged from contacts with the local population. Employers may also recruit migrants as a way of accessing networks of workers who can be hired for short periods at short notice. Such factors may support efficiency and resilience, but if so, at what cost to migrant workers themselves?

In principle, employers have a number of options when they perceive staff shortages (Ruhs & Anderson, 2010): they can (i) raise wages and/or improve working conditions to attract more applicants from the domestic labour market (including from other sectors) and/or expand the working hours of existing staff, which may require intensified recruitment efforts and investments in training or up-skilling, respectively. Alternatively, employers can (ii) reduce the labour intensity of the production process by increasing the capital and/or technology intensity; (iii) relocate production to countries with lower labour costs; (iv) redirect the business towards less labour-intensive commodities and services; and (v) employ more migrant workers. While some of these options will not be available in certain sectors or occupations, several options are typically also available in essential services: for example, the pandemic may have reinforced a trend among US agricultural producers towards greater mechanisation (Martin, 2020). In general, the recruitment of migrant workers is not the only possible response to a staff shortage. Nor is it necessarily the best, from the point of view of employers or states.

Accordingly, while migrants often represent a substantial share of the workforce in essential services, these shares vary significantly between countries (Fasani & Mazza, 2020; OECD, 2020a). Such cross-country variations for specific sectors and occupations partly reflect country-specific ‘system effects’. They arise from the institutional and regulatory frameworks of the labour market, interlinkages with global supply chains and wider public policies such as migration, welfare, housing as well as education and training policies (e.g. Afonso & Devitt, 2016; Ruhs & Anderson, 2010; Wright, 2012). Often beyond the control of individual employers and workers, these system effects may be significantly (albeit not exclusively) influenced by the state and can ‘produce’ domestic labour shortages. Due to system effects, labour migration policy interacts with labour market policy and wider economic and social policies and institutions. Cross-country variation in reliance on migrant workers is related in significant part to differences in institutional and regulatory frameworks, and the same is likely to be the case for the variation in resilience strategies for the provision of essential services.

Finally, it is important to recognise that the role migrants can and should play in addressing perceived labour and skills shortages is a deeply political issue. Whether the best answer is more immigration, higher wages, greater mechanisation, or another alternative critically depends on whose interests policy is meant to serve, and how competing interests (between employers, workers from the domestic labour market, migrants, employment and recruitment agencies etc.) are evaluated and managed. In these debates it is often very difficult for the interests of migrants to be properly represented. For analyses and debates about the role of migrants in shaping systemic resilience, this implies a politics of systemic resilience just as there is a politics of labour and skills shortages. This politics will influence how resilience is defined and measured, how it is governed, and – at least to some extent – which resilience strategies are given priority. Empirical research in this context therefore needs to consider carefully definitions and measurement issues and account for the strong role of policies and institutions (Section 5).

## The concept of systemic resilience

In the broadest sense, systemic resilience can be defined as a system’s capacity to continue functioning given external shocks or changes (Martin-Breen & Anderies, 2011). Early use of this notion was made in ecology, where systemic resilience captures the capacity of an ecosystem to recover from a shock, returning to the status quo ante. A similar idea of systemic resilience has also long been used in physics and engineering. In a social science context, systemic resilience has been used with a key modification: after a shock, a resilient system continues to function but might end up in a state that is quite different from the status quo ante.

This follows from the nature of systems in social science, which are in general made up by (groups of) persons who are connected through relationships such that they form some collective entity – for example a household, a firm, a city, an industrial sector, regions or countries. Such systems are typically in a process of change also in the absence of shocks (e.g. Robinson & Carson, 2016). In addition, social systems are adaptive: they can self-organise and find new modes of operation in order to continue functioning (Martin-Breen & Anderies, 2011). These considerations lead to defining systemic resilience here as the ability to withstand, recover from, and adapt to unexpected external shocks (e.g. OECD, 2020b).

**Fig. 1:**
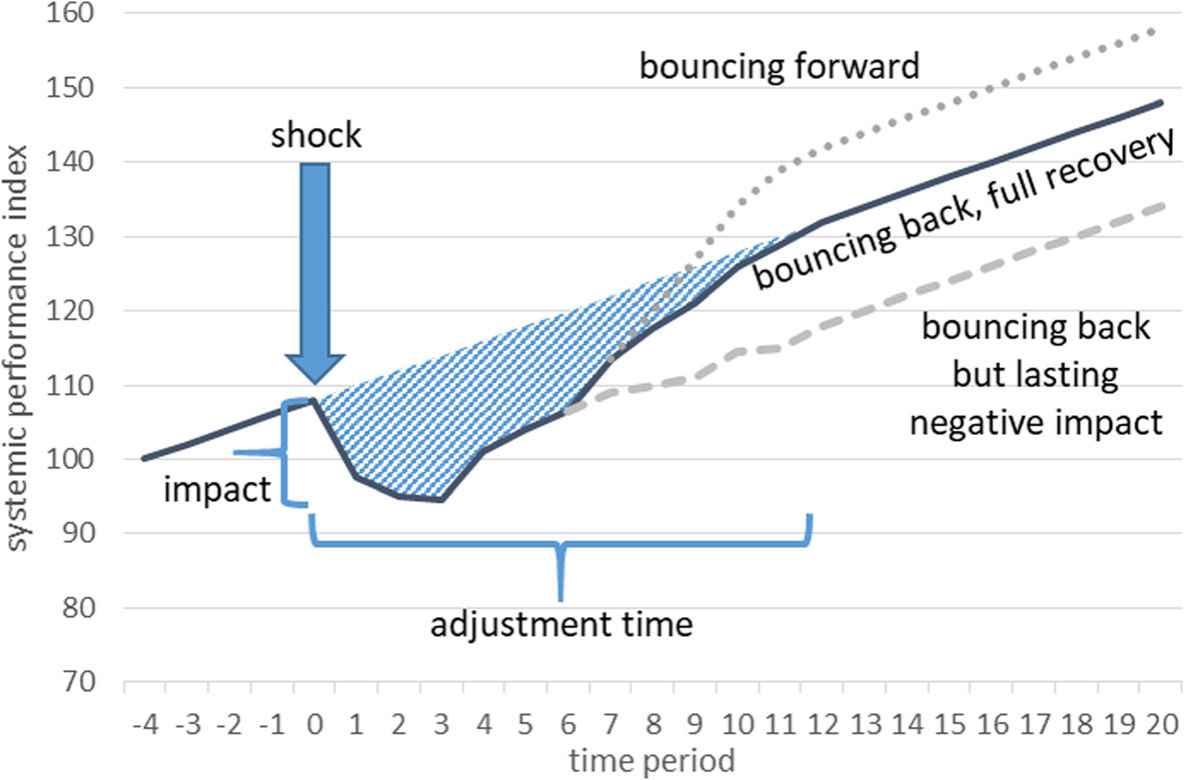
Illustration of systemic resilience

Key elements in the definition of systemic resilience are shown in Fig. 1. It depicts some social system in the process of steadily rising ‘performance’, captured by an index. When a shock hits, systemic performance might initially decline substantially, down to a point (‘impact’). Over time, however, the performance of a resilient system bounces back. In the baseline scenario (solid dark line), performance fully recovers, eventually proceeding as if the shock had never occurred. In another scenario, the recovery is only partial. In a third scenario, the adaptation by the system not only leads to recovery but also creates lasting benefits, so that performance bounces forward.

Figure 1 hints at how systemic resilience might be measured, a precondition for comparing systems in terms of resilience. More resilient systems would normally exhibit a smaller impact from a given shock and/or a shorter adjustment time than less resilient systems. The two indicators may be combined into a single measure (the shaded area in Fig. 1) decribing the overall loss of performance following the shock, and a lower overall loss would indicate a more resilient system. A system could therefore be more resilient than another even when it exhibited a greater impact, as this can be outweighed by a shorter adjustment time, and vice versa.

While some contributions to the literature highlight the overall loss as a measure (e.g. Linkov & Trump, 2019), others emphasise the lack of a generally agreed method for measuring systemic resilience (e.g. Martin & Sunley, 2015). In any case, the magnitudes of the ‘impact’ and ‘adjustment time’ will critically depend on how the ‘performance’ of the system is defined and measured in the first place. It already emerges from Fig. 1 that determining the adjustment time may be far from obvious in many scenarios. Like defining and measuring resilience, these are also political issues.

The notion of systemic resilience has been applied in numerous disciplines of social science, often using more context-specific definitions (Brand & Jax, 2007). This includes development studies, economics, geography, management science and public health – while migration studies have essentially remained disconnected from this work, both in the academic literature and in policy-oriented publications. Nevertheless, the research on systemic resilience across disciplines has produced a number of insights that appear highly relevant for the resilience of essential services and for the role of migrant labour in this context.

These insights can be grouped under three broad themes: flexibility, networks, and policies. Intuitively, a system may be hard to break (resilience) if it bends (flexibility) and if connections between its fibres are strong (networks). Policies or management have an indirect effect, by creating conditions that can be more or less conducive to systemic resilience. All three themes include factors that limit the impact of a shock as well as factors that shorten the adjustment time, and factors might often contribute in both ways.

Factors shaping the flexibility of a system have been identified at various levels. In firms or institutions, individual employees can find workarounds and alter their work schedules, but also organisational structures and corporate strategies can be modified in response to shocks (Eichhorst et al., 2010; Woods, 2006). As part of such adjustments, production processes may change to a substantial degree (depending on their ‘transformability’): by substituting for inputs, e.g. in terms of materials or suppliers, as well as outputs, e.g. remodelling the product or targeting different markets. The process itself may be reorganised or entirely replaced by another process. Where redundant options are in place for a part of the system, its performance will be less dependent on this part functioning normally (Martin & Sunley, 2015). Buffers, for example in the form of excess or surge capacity, can play a similar role (Hynes, 2020). It is more likely that key functions of systems can be sustained whenever a diversity of mechanisms is available to deliver them (Martin-Breen & Anderies, 2011). However, redundancies and excess capacity create additional costs (Kamalahmadi & Parast, 2016), which highlights a trade-off between flexibility and cost-minimisation.

The strength of networks, or social capital, can equally contribute to systemic resilience in a variety of ways. The resilience of a hospital, for example, likely depends on the commitment and team spirit of its staff, which affects the extent of burden sharing, information sharing and peer learning (Kruk et al., 2015). After team building, groups adapt better to new challenges, according to experimental evidence (Randall et al., 2011). The replacement of staff, additional recruitment as well as mobility and redeployment may all be organised through networks. During crises, networks can become an important resource: currently unaffected nodes of the network can come to the aid of the most affected nodes, thereby functioning as insurance for each other. Such structures can be supported through a common goal or reciprocity (Ostrom, 2003) but may have to be sufficiently large or modular to make collective failures unlikely. By enabling rapid temporary increases in staff levels, for example, networks also contribute to flexibility.

Finally, there is an important role for policies (or management) because they shape institutional structures and therefore the conditions for any efforts to keep systems functioning despite shocks (e.g. Briguglio et al., 2009; OECD, 2012; Thomas et al., 2021). Policies that set rigid standards and regulations can limit the scope for flexibility, and centralised institutions can block the operations of decentralised networks. At the same time, policies can also enforce certain preparations and crisis interventions that are individually costly but collectively beneficial. As an extreme example, firms may be mandated to start producing certain medical equipment. More commonly, however, policies and management affect systemic resilience by creating incentives and disincentives, albeit not necessarily intended. The significant differences between systems across countries attest to the long-run implications of different policies.

## Introducing systemic resilience into the analysis and regulation of labour migration

In order to incorporate the resilient provision of essential goods and services as an important policy goal, research and policy on migration need to be adapted. This requires rethinking the design of labour migration policies as well as wider public policies, and the framework for evaluating the impact of migrant labour. Some first implications are discussed in this section.

### Shifting the focus to transnational systems

In order to investigate systemic resilience, systems need to be the unit of reference. A holistic approach to entire systems has to go beyond the common practice of investigating the impact and policies of labour migration in terms of specific skill levels or occupations. This narrow focus ignores the systems that occupations are embedded in, meaning that effects on the functioning and the resilience of the systems go unnoticed.

Similarly, the impact of migration and migration policies is often evaluated in the context of one particular country. Even research on how systems shape the demand for migrant labour has mainly focused on the role of *national* institutions and policies. Attention to resilience requires broadening the analysis of systems to consider for example transnational supply chains, and how these interact with national policies. As observed for medical products during the Covid-19 pandemic, domestic production and supply chains, trade in intermediate or final products, and associated supply chains abroad can all be part of the systems behind essential goods. Typically, several economic sectors are involved in a supply chain – for example agriculture, food processing, transport, wholesale and retail trade are all involved in the supply chain for food. Supply chains may rely on migrant workers in various roles, both domestically and abroad. For example, migrants in Malaysia appear to produce much of the global supply of the medical gloves that are necessary to the provision of both health and social care across the world (Khadka, 2020). Mechanisms for resilience also often work across borders and demand international collaboration (Hynes, 2020). This raises important questions about when national borders enhance and when they undermine resilience.

For labour immigration policies, consideration of systemic resilience reinforces the relevance of the situation in migrants’ origin countries. Migration policies of destination countries reflect their own national interest, but the resilience of essential services requires a more transnational approach to impact analysis and policy-making on labour migration than is typically the case. Currently, most high-income countries’ labour immigration policies are discussed and designed unilaterally with little (if any) consideration of the consequences for migrants’ countries of origin (Ruhs, 2013). More cooperative labour migration policies such as bilateral or multilateral migration schemes based on agreed standards and communication channels with origin countries could improve systemic resilience. For example, in a crisis, destination countries could draw on such schemes to quickly increase the supply of migrant labour with certain skills or work experience, such as nurses or returning seasonal workers.

If resilience strategies in destination countries lead to a greater demand for migrant labour, this can threaten resilience in origin countries. Global care chains, e.g. nurses from Europe going to the US while nurses from the Philippines go to Europe, are an example of how emigration can create shortages in migrants’ countries of origin (Yeates, 2010). By seeking to increase resilience of their essential services, destination countries risk ‘exporting’ vulnerabilities to origin countries. If on the other hand the resilience strategies in destination countries lead to lower demand for migrant labour, this could reduce remittance flows to origin countries. When this happens as a crisis response, e.g. due to border closures for seasonal migrants, an economic contraction in destination countries can be passed on to origin countries. In case only the origin country experiences a crisis, temporary return of some emigrants could make an important contribution to the systemic resilience of its essential services. More generally, greater global resilience requires that systemic resilience also becomes a policy goal in origin countries and is supported by destination countries.

### From short term to long term

Considering systemic resilience necessarily leads to a more expansive temporal frame: most analyses and policy debates on migration are focused on the short term, but a concern with systemic resilience demands a longer-term view. While there has been a considerable increase in recent years in studies that analyse the long-term consequences of migration (e.g. Sequeira et al., 2020), they have not, to the best of our knowledge, looked at how migrants shape systemic resilience.

Drawing lessons from past events, including Covid-19, requires us to be conscious of future ‘extreme’ contingencies in current decision-making, thinking about long-term processes and short-term system dynamics concurrently (Dunn Cavelty et al., 2015). Since extreme events arise rarely, attention to the medium and long term is a precondition for systemic resilience, in a trade-off against pressures to focus on the short term. For employers, short-term efficiency demands using all inputs to maximum effect, while resilience may require keeping reserves and leaving room for adjustment. For politicians in democracies, elections create very strong incentives to pursue short-term objectives, while resilience demands looking beyond the electoral cycle. Short-term bias has also been debated in the context of sustainability, which similarly relies on thinking with a long time horizon (Harding, 2006). However, systemic resilience additionally requires an acute awareness of – even unquantifiable – uncertainty and risk.

The shift in temporal framing will also need to accommodate the temporalities of migrant decision-making. It is not new to note that people may temporarily tolerate harsh working conditions for the promise of a better future. Research has examined the imbrication of temporalities, public policies and migratory decision-making in certain contexts (see e.g. Griffiths et al., 2013 for a review and more recently Mavroudi et al., 2017). This work has explored subjective experiences of time including how this is shaped by immigration policies. However, it has so far not been connected to other public policies, and the focus has been on personal and familial endurance rather than systemic resilience.

### A new politics of labour immigration

Given the inherently political nature of migration, taking systemic resilience seriously as a policy goal, and making the above-described changes, will require a new politics of labour migration especially with regards to what is currently termed medium and low-skilled labour migration. This new politics will need to facilitate and encourage policy debates and collective decision-making that embrace some difficult trade-offs for societies in destination countries: let wages and prices in social care rise or recruit migrants to fill shortages at existing wages? Finance the excess capacities in healthcare that are needed for emergency situations or minimise costs and count on crisis response policies? Rely on migrant workers to maintain domestic food production or allow for more imports of food?

In a post-Covid-19 world, debates and decisions on these trade-offs need to go beyond the conventional short-term focus on efficiency (what are the economic costs and benefits of immigration today?), welfare (what are the fiscal effects of migrant workers in a particular year?) and distribution (what are the impacts of migrant workers on different groups of citizens?) and consider longer-term concerns including systemic resilience in the provision of essential goods and services. This will entail deciding on how much systemic resilience is ultimately desired, as there may be a point where the short-term costs of raising resilience further outweigh the long-term benefits. It will also require intensified international collaboration in the context of growing pressures towards protectionism. Given the debates about how essential services fared during the Covid-19 pandemic and the role played by migrants in this context, there may now be greater scope for both debating these difficult trade-offs and engaging in greater international cooperation.

### From protecting the employment of citizens^1^ to protecting the provision of essential services

Both public debates about international migration and the design of labour migration policies typically put the employment prospects of citizens centre stage. Accordingly, impact assessments of migration usually examine how the wages and employment of citizens are affected by immigration (e.g. Migration Advisory Committee, 2012). In public debates, the relationship between migrants and citizens is often approached through an ‘us versus them’ frame (e.g. Anderson, 2013). Labour migration policies typically include a ‘labour market test’ as a standard feature: before employers can obtain a work permit for a migrant worker, they need to demonstrate that the position could not be filled from the domestic labour market (although exemptions often apply to high-skilled migrant workers). These approaches and policies serve the political rationale to prioritise the citizens’ access to the national labour market and thereby protect their employment prospects.

In contrast, protecting and enhancing the resilience of the provision of essential goods and services is a different policy goal that might compete with the protection of citizens’ employment prospects: the need to ensure the stable provision of essential services can outweigh distributional concerns and efficiency considerations. As essential services are fundamental for basic social functioning and people’s survival, the ‘means’ of providing them (through some combination of citizens and migrant workers) are of secondary importance compared to the ‘ends’ (maintaining the provision). While this might lead to lower barriers for the recruitment of migrants, prioritising systemic resilience could also lead to deteriorating working conditions and greater exploitation among migrant workers in essential services, justified by the need not to endanger the functioning of essential services.

Covid-19 exposed the often precarious employment conditions of ‘key workers’ who work in contexts that increase their vulnerability to infection and are often employed on temporary contracts with limited rights (e.g. Nivorozhkin & Poeschel, 2021). Trade unions and migrant activists protested that while some work was recognised as ‘essential’, workers themselves were treated as ‘disposable’ (Coleman, 2020; Dias-Abey, 2020). Many commentators called for stronger rights and greater security for existing migrants in essential services, as well as more legal pathways and opportunities for future migrants to work in these occupations. These debates raise the important broader question of how the socio-economic vulnerabilities of migrants employed under restricted rights shape their role in facilitating systemic resilience.

## A new agenda for comparative migration research: how do international migrants shape systemic resilience?

There has not yet been research on how international migration affects the systemic resilience of essential services. The Covid-19 pandemic has highlighted the contributions migrants make to the provision of essential economic activities and services, both in countries where these goods and services are consumed/accessed and along global supply chains. Migrants’ jobs and their behaviour in these jobs may well differ from those of citizens in ways that matter for resilience. For example, recalling the features of resilient systems identified in Section 3, migrant workers might be especially flexible, or conversely, immigration requirements might reduce flexibility. Similarly, migrants’ social capital may play roles in transnational networks that are relevant during crises. Thus, the employment of migrants could affect both the magnitude of the decline in performance of a system following a shock as well as the adjustment time (see Fig. 1).

Examining the link between migrants and systemic resilience requires *comparative* research at several levels. Migration is only one among various factors affecting resilience. A comparative approach is needed to disentangle the effects of migrant workers from the impacts of other factors, especially the characteristics of citizens employed in essential services and the effects of policies and institutions. For example, the resilience of the provision of health services in a particular country is likely to be shaped by its general systemic characteristics including how work is organised, regulated and has been prepared for a crisis. At the same time, migrant workers can affect resilience in various direct and indirect ways, many of which might not be obvious and need to be ‘discovered’ through research and experience. Therefore, we do not attempt to provide a list of concrete research questions for comparative analysis. Instead, we identify three types of comparative research set-ups that might shed light on how migrants shape systemic resilience of essential services: comparisons between migrants and citizens within one system (e.g. a system for providing social care in a particular country), comparisons across systems (e.g. across systems for providing care in different countries), and comparisons of the politics of resilience strategies.

### Comparing migrants and citizens employed within the same system

The first type of comparative research explores effects on systemic resilience that are specific to migrants and do not arise for citizens even when working in the same jobs. There are a number of reasons why such migrant-specific effects can arise, including the migratory process: in the case of temporary migration, labour supply is conditional on ongoing possibilities to migrate. In the case of longer-term migration, it is conditional on the renewal of residence and work permits. The Covid-19 pandemic highlighted that these conditions can translate into migrant-specific vulnerabilities for systemic resilience: border closures prevented migration, quarantine delayed and discouraged migration, and status renewals became uncertain. Ad-hoc policies mitigated these problems but did not fully resolve them (Section 1). Migrants might also choose to stay away from the destination country or to leave the country, as was observed for some migrants working in social care in Germany (Safuta & Noack, 2020).

However, the possibility to recruit migrants from abroad can also be an important source of resilience, particularly if the crisis is national or regional rather than global. From the perspective of destination countries when domestic sources of labour and skills are under strain during a crisis, migrants can be a temporary ‘back-up’ from abroad, provided the origin country is not as badly affected as the destination country. Such temporary support can be arranged through cross-border networks (formal or informal), and migrants are well-positioned to create cross-border networks. In the context of established bilateral or multilateral migration schemes, recruitment of migrants for essential services could be organised especially quickly. This could involve fast-track procedures for recognising or adapting migrants’ foreign qualifications, in order to avoid lengthy delays due to missing licenses or permissions.

In the destination country, unlike citizens, migrants face constraints imposed by immigration controls, and this could affect systemic resilience. Work and residence permits are often tied to a particular employer, which rules out changing jobs and equates losing the job with losing the right to residence. Therefore, migrants are often especially committed to their current job even under the difficult conditions of a crisis, thereby contributing to systemic resilience. As discussed earlier, employment restrictions can make migrants much more exploitable than citizens, and some employers might lower employment conditions in a way that has adverse impacts on systemic resilience. Similarly, migrants’ access to local healthcare and child care is often restricted (especially for irregular migrants), which can undermine their ability to work. Finally, medium and low-skilled migrants often have limited rights to family reunification. Migrants living more often alone than citizens may be advantageous in a pandemic but lack of family support could be detrimental in other crises.

In addition to the process of migration and the related effects of migration controls, the individual migration experience could matter for systemic resilience. The fact that migrants needed to adapt to the new environment in the destination country might mean that they adapt to a crisis situation comparatively easily. If they previously worked in similar roles in the origin country, this might help them develop workarounds: migrants might think more ‘out of the box’ because they are aware of different approaches. Diversity of work teams in terms of prior experiences, talents or training has been linked to stronger team performance (e.g. Horwitz & Horwitz, 2007).

Fourth, migrants’ average age and health characteristics may differ from those of citizens. Emigration often takes place at a young age and so migrants might on average be comparatively young while they work in the destination country. This may matter for resilience, as was evidenced in the Covid-19 pandemic when young persons faced a lower risk of falling ill (Promislow, 2020). It may also affect their adaptability. In addition, migrants might self-select based on the strength of their health (e.g. Lariscy et al., 2015). A ‘healthy-migrant effect’ would play a role for systemic resilience, notably during pandemics. If, on the other hand, migrant workers are poorly housed, they can face an especially high risk of infection (Koh, 2020).

For most of these differences between migrants and citizens, the implications for resilience can be studied within a single system, as long as it employs both migrants and citizens in substantial numbers. Using observations on both groups over time (at least before and after a shock), one can examine empirically how migrant workers fared, behaved, and affected the resilience of the provision of a particular essential service compared with citizens. This also offers an opportunity to examine similarities and differences with internal migrants, whose mobilities may be facilitated or constrained using non-immigration policies. These national contexts can also interact with global supply chains.

### Comparing migrants’ roles across systems

The role of migrants in shaping systemic resilience may be largely determined by policies, regulations and institutions that differ between particular essential services and between countries (Section 2). This means that an effect ascribed to migrant labour might reflect the structure of an essential service – for example, a structure that relies on the strong involvement of migrant labour. In order to reach reliable empirical conclusions for research and policy, it will therefore be important to distinguish between effects specific to migrants (as discussed above) and effects from certain roles that migrants play in the specific institutional structure of an essential service. In other words, we need to study comparatively how different institutions and policies (such as different institutional designs of the care system) shape the resilience of the provision of an essential service, and what this means for the role of migrants.

For example, if national immigration controls allow limiting a migrant’s employment to specific parts of the country (a policy that varies considerably across countries), labour migration policy can be used to assign migrant health professionals to positions in rural areas (e.g. Hagopian et al., 2003) – and a sufficient presence of health professionals in rural areas may be important for the systemic resilience of healthcare. However, in other contexts such a policy might undermine resilience: migrants tend to be more geographically mobile within the destination country (Boman, 2011), and such flexibility can become particularly important during a crisis.

In systems for essential services that rely on flexible labour markets, migrants might work disproportionately in roles with low pay, non-standard working hours, and limited contracts. If the existence of these jobs affects systemic resilience (either positively through e.g. enhanced flexibility, or negatively through e.g. poor working conditions), migrant workers impact on resilience. Research has found that the availability of migrant labour can affect the skills mix and use of capital in production and may in some cases expand the number of labour-intensive jobs (e.g. Lewis, 2011). This suggests that migrants play specific roles in systems and can affect systemic resilience by influencing some of the institutional characteristics of the system itself.

When analysing the role of migrants within different systems for providing an essential good or service, it is important to consider the transnational dimension. The resilience of a supply chain is often dependent on laws and policies in a number of different jurisdictions, including the migration and labour policies of countries other than the country of final destination. Thus importing certain crops for example does not per se reduce reliance on migrant labour systemically, although it might alleviate national political concerns about this reliance by effectively outsourcing it to other countries. Such interdependencies through supply chains highlight the need for international collaboration in order to increase systemic resilience.

The following questions thus arise for comparative research on the role of migrants across countries with different systems for providing an essential service: how do systems that strongly rely on migrant labour perform in terms of systemic resilience compared with systems that make much more limited use of migrant labour? In other words, what does it mean for resilience when an essential service has come to rely heavily on migrant labour, compared with systems that have moved towards greater mechanisation, employment of citizens at rising wages, or reliance on imports (and thus greater use of migrants in the supply chain abroad)? Such comparative research can draw on cross-country differences in the reliance of essential services on migrant labour, differentiating by migrants’ roles and relating this to observed differences in resilience.

### Comparing the choice and determinants of resilience strategies across systems

Building on research that assesses the effects of migrants on resilience within and across systems, it is important to ask whether and why particular resilience strategies are more or less likely to be adopted in different national contexts. We need to understand the factors that constrain and influence the (non-adoption of) particular resilience strategies by national governments, which requires comparative political and institutional analysis. It also requires that we examine how resilience strategies in particular countries are related to the institutions and policies of other countries. For example, as Covid-19 revealed, facilitating labour immigration during a global pandemic requires that sending states facilitate labour emigration.

As discussed above, policy responses to Covid-19 could include a change in the use of migrant labour within a given system (i.e. without changing the broader institutional and policy framework of the system) or a ‘regime switch’ to a different system (e.g. a change to a social care system with different institutional features that are more supportive of systemic resilience). The political choice among these strategies can be influenced by a range of factors. A first obvious factor are the material interests of different groups and the effects of different resilience strategies on them. A change in the use of migrant workers (e.g. in their numbers, the roles they fill, and/or the rights they are given) will create costs and benefits for different political actors and groups, and the same holds for a ‘regime change’ such as a significant change in labour market regulations and/or associated welfare policies. The distributional and other consequences of different strategies for improving systemic resilience can be expected to play an important role in whether or not particular policies are adopted. Similarly, stakeholders will seek to influence what exactly qualifies as an essential good or service. A comparison of official lists of essential services published by Italy, Spain and the United States, for example, reveals a strong overlap but also notable differences (Nivorozhkin & Poeschel, 2021).

Effects and path dependencies of institutions and associated social norms are also likely to matter for the choice of resilience strategy. A long tradition of regulating labour markets and societal norms that value high degrees of socio-economic security, solidarity, and social protection might significantly constrain an increase in the reliance on migrant workers on temporary contracts, to make the provision of a particular essential service more flexible and thus more resilient. Regime change will depend on the characteristics and rigidities in the prevailing institutions and norms. Again, this may vary across countries and thus play an important role in explaining the adoption of different resilience strategies.

Third, public attitudes to labour migration and concrete resilience strategies are likely to matter as well. An increase in the use of migrant labour in essential services may well lead to a rise in low-skilled immigration, as many essential jobs are in low-wage occupations. We know from existing research that public attitudes to labour migration critically depend on the skill and perceived contributions of the migrant, and in most countries they are much more positive to higher-skilled than to low-skilled migration (e.g. Hainmueller & Hopkins, 2014). An important question for research is whether and how Covid-19 has impacted on these attitudes, and specifically whether the recent apparent public appreciation of key workers in many countries has improved public attitudes towards lower-skilled migrant workers doing essential jobs (Dražanová, 2020). Comparative research could explore whether and why the impact of Covid-19 on public attitudes differs across countries, as this may have significant consequences for the resilience strategies considered most acceptable by policymakers.

## Conclusion

The Covid-19 pandemic has raised new and urgent research and policy questions about the factors that shape the resilience of essential services to major shocks, especially with regards to food and agriculture, health services, and social care. There is little doubt that epidemics and pandemics will happen again but their effects will depend critically on actions to improve the resilience of the provision of essential economic activities and services. As migrants often represent substantial shares of ‘key workers’ albeit to different degrees across countries, it is important to analyse their role in shaping systemic resilience in different institutional contexts. Existing research on the effects and regulation of labour migration has largely focused on ‘efficiency’ (costs and benefits of immigration) and ‘distribution’ (effects of immigration on different groups of people) as key outcomes of interest without paying significant attention to issues related to systemic resilience.

This paper provides a basic framework and conceptual building blocks for analyses and policy debates about the role of migrant workers in the provision and resilience of essential services. Bringing together key insights from research on labour migration (specifically the role of migrant workers in addressing labour and skills shortages) and work on systemic resilience in other disciplines, we explain why and how a concern for the resilience of essential services should make us rethink how the impacts of migrant workers are assessed and how labour migration and related public policies are designed. Taking systemic resilience seriously as a policy goal requires us to shift the focus of analysis and policy debates from the role of migrants in specific occupations and sectors in particular countries to transnational systems of production and service provision. There is a need for greater attention to medium and long-term effects of labour migration as well as to the role of international supply chains and international collaboration. This in turn might result in a reduction in the relative importance typically attached to the protection of citizens in labour migration policy-making. Analyses of migrants and systemic resilience also demand greater consideration of the interlinkages between migration and other public policy areas which, among other things, requires a multidisciplinary approach.

Some of these changes – such as the greater emphasis on the long term, the analysis of migrants’ effects along the entire transnational supply chain, and linking migration policies with other public policies – would help address what in our view have been long-standing gaps and deficiencies in research and policy debates on labour migration. The new research agenda we propose, to help understand the link between migrants and systemic resilience, requires a *comparative* approach at several levels, to disentangle the effects of migrant workers on systemic resilience from the effects of policies or institutions. We need comparative analysis of how migrants and citizens affect systemic resilience *within given systems*, and of how and why resilience varies *across systems* and *across countries* characterised by different institutional and policy frameworks and, therefore, different degrees of reliance on and roles for migrant labour. The comparative institutional analysis also needs to pay attention to politics and systemic changes over time, considering whether, how, and why different systems change, including but not limited to their use of migrant labour.

The new analysis of the role of migrant labour in shaping systemic resilience across institutional contexts that we propose in this paper is closely connected to, but different from, discussions about restrictions on low-skilled labour migration and the exploitation of migrants in low-waged jobs. As we point out in Section 5, analysing how migrants can shape systemic resilience requires a consideration of how pre-crisis policies and institutions, the crisis itself and any policy responses to it affect migrants themselves. This type of analysis can produce important and, we would argue, urgently needed insights on the many inter-relationships and trade-offs between facilitating greater systemic resilience through particular uses of migrant labour (e.g. through the enhanced flexibility that migrants on temporary contracts may provide) and the employment conditions and socio-economic security of migrants. We thus consider our rethink and approach as encompassing rather than only complementary to analysis and debates about the effects of the pandemic and policy responses on migrants themselves.

While our analysis is motivated by Covid-19 and the role of migrants in shaping systemic resilience to the current pandemic and similar future *health shocks*, the paper is also relevant for shocks with similar characteristics as the current pandemic, especially with regards to its transnational reach, relative suddenness of onset and impact, and threat to human health. Examples of shocks that may share these characteristics with Covid-19 and other pandemic shocks include environmental shocks with transnational reach (e.g. major earthquakes, tsunamis, and extreme weather events), human-made disasters (e.g. nuclear meltdowns), and failures of international infrastructure (e.g. protracted breakdowns of power networks or pipelines). These scenarios can all create challenges for essential services to continue operating in a suddenly more difficult and somewhat dangerous environment. Other types of shocks such as financial and economic shocks raise different issues (e.g. Strauss-Kahn, 2020) and therefore require different policy responses with their own specific implications for the role of migrant labour in supporting systemic resilience.

One of the criticisms of resilience-thinking is that it takes socio-economic problems as matters of fact that must be adjusted to rather than challenges that can and must be tackled in their own terms. We recognise that it is often important to understand structural causes and not simply ameliorate symptoms. However, shocks do occur and some are outside of human control. In a globalised and interconnected world, it is increasingly likely that these shocks are themselves globally interconnected. Building systemic resilience therefore entails not cementing the status quo but preparing for uncertainties in the future.

## Data Availability

not applicable.

## Abbreviations

Covid-19: Coronavirus disease
EU: European Union
IOM: International Organization for Migration
OECD: Organisation for Economic Development and Co-operation

## Footnotes

1 While ‘citizens’ and ‘migrants’ are typically counterposed in public debate in many states it over-simplifies and overlooks the fact that many residents may not be citizens yet have the right to unrestricted access to the national labour market (e.g. non-citizens with permanent residence status).
